# Somatic Development and Some Indices of Lipid Metabolism in 11-year-old Children Born with Extremely Low Birth Weight (<1000 G) (long-term Cohort Study)

**DOI:** 10.34763/devperiodmed.20172104.361368

**Published:** 2018-01-02

**Authors:** Nina Mól, Magdalena Zasada, Małgorzata Klimek, Przemko Kwinta

**Affiliations:** 1 Department of Pediatrics, Institute of Pediatrics, Jagiellonian University Medical College, Krakow, Poland

**Keywords:** prematurity, extremely-low-birthweight, somatic development, wcześniactwo, ekstremalnie mała masa urodzeniowa, rozwój somatyczny

## Abstract

**Aim:**

To continue the prospective follow-up cohort study on the somatic development and lipid metabolic parameters of 11-year-old-children born with extremely low birth weight and to compare them with the results obtained in the study of 7-year-old children from the same group.

**Material and methods:**

204 newborns with birth weight ≤1000 g were born in the Malopolska voivodship between 1.09.2002 and 31.08.2004. From this group 115 (56%) children died during infancy and 89 (44%) infants survived. At the age of 7 years 81 (91%) of the children from this group were examined. At the age of 11 years investigations were carried out in 62 (75%) of the children, while 19 (26%) were lost to follow-up. All the children underwent anthropometric measurements. Moreover, the lipid profile (serum total cholesterol, triglycerides, HDL-cholesterol, LDL cholesterol) was evaluated.

The control group consisted of 36 children born at term chosen randomly from the general population and matched with regard to age and sex.

**Results:**

Children born with extremely low birth weight were generally smaller than their peers. At 7 years, they were shorter (113.75 cm(-0.72) vs. 124.52 cm(0.53)), lighter (19.47 kg(- 1.12)vs.25.23 kg(0.39)), had a smaller head circumference (49.81 cm(-2.19) vs.52.5 cm(-0.377)), waist circumference (50.14 cm(-0.83) vs.55.45 cm (0.34)), mid-upper arm circumference (17.51 cm vs. 19.29 cm), skinfold thickness (0.76cm(-0.817) vs.0.92cm (-0.19)) and body mass index (14.5 kg/m^2^(-0.99)vs.16.16 kg/m^2^(0.12)) expressed both as absolute values and z-score values compared to the control group.

At 11 years old, the height (141.7 cm(-0.368) vs. 146.26 cm(0.65)), weight (33.88 kg (-0.59)vs.40.45 kg(0.66)), head circumference (51.37 cm(-2.05)vs.54.02 cm(-0.33)), waist circumference (61.7 cm (0.26) vs.67.84 cm( 1.06)), mid-upper arm circumference (20.95 cm vs. 22.85 cm), skinfold thickness (1.17 cm(-0.25)vs.1.68 cm(0.78)) and body mass index (16.74 kg/m^2^(-0.62) vs. 18.72 kg/m^2^(0.36)) expressed both as absolute values and z-score values were still lower in children born with extremely low birthweight than in the control group. However, their gains over the time period between 7 and 11 years were comparable to their born-at-term peers in all the measured anthropometric parameters. There were no statistically evident differences in the indices of lipid metabolism.

**Conclusions:**

Preterm children with extremely low birth weight (<1000 g) are at an increased risk of growth failure. Once they reach teenage years they are shorter and lighter than their age- and sex-matched born-at-term peers. They also have smaller heads. In our study we did not find statistically evident differences between the investigated and control group in lipid indices. There is a need for longitudinal studies to observe somatic, mental and metabolic development in order to organize multidisciplinary holistic medical care for them.

## Streszczenie

**Cel:**

Ocena w ramach prospektywnego badania kohortowego parametrów rozwoju somatycznego oraz niektórych parametrów przemiany lipidowej u 11-latków urodzonych z ekstremalnie małą masą ciała (<1000 g). Porównanie wyników badań 11-latków z wynikami uzyskanymi w badaniach przeprowadzonych w 7 roku życia u tych samych dzieci.

**Materiał i metody:**

W regionie przeprowadzonych badań w Małopolsce w okresie między 1.09.2002 and 31.08.2004 urodziło się 204 noworodków z ekstremalnie małą masą urodzeniową (<1000 g). Z grupy 204 noworodków zmarło w okresie niemowlęcym115 (56%). Przeżyło 89 (44%). Do długofalowych badań w 7 roku życia zgłosiło się z 81 dzieci (91%), nie zgłosiło się 8 (9%). Do badań 11 latków zgłosiło się z tej kohorty 62 dzieci (75%), nie zgłosiło się 19 (26%). U wszystkich tych dzieci, stanowiących badaną kohortę, wykonano badania antropometryczne oraz oceniono profil lipidowy (stężenie całkowite cholesterolu, TGL, LDL, HDL). Wyniki porównano z wynikami dzieci z grupy kontrolnej urodzonych o czasie (n=36) z prawidłową masą ciała.

**Wyniki:**

Dzieci urodzone z ekstremalnie małą masą urodzeniową były mniejsze niż dzieci w grupie kontrolnej. W wieku 7 lat były niższe (113.75 cm(-0.72)vs.124.52 cm(0.53)), lżejsze (19.47 kg(-1.12) vs.25.23 kg(0.39)), miały mniejszy obwód głowy (49.81 cm(-2.19)vs.52.5 cm(-0.377)), obwód pasa (50.14 cm(-0.83)vs.55.45 cm (0.34)), obwód ramienia (17.51 cm vs. 19.29 cm), grubość fałdu skórnego (0.76 cm (-0.817)vs.0.92 cm(-0.19)) oraz wskaźnik masy ciała (14.5 kg/m^2^(-0.99)vs.16.16 kg/m^2^(0.12)) wyrażone zarówno jako wartości całkowite jak i z-score. W wieku 11 lat wysokość (141.7 cm(-0.368)vs.146.26 cm(0.65)), masa ciała (33.88 kg(-0.59)vs.40.45 kg(0.66)), obwód głowy (51.37 cm(-2.05)vs.54.02cm(-0.33)), obwód pasa (61.7cm(0.26)vs.67.84cm(1.06)), obwód ramienia (20.95 cm vs. 22.85 cm), grubość fałdu skórnego (1.17 cm(-0.25)vs.1.68 cm(0.78)) oraz wskaźnik masy ciała (16.74 kg/m^2^(-0.62)vs.18.72 kg/m^2^(0.36)) wyrażone zarówno jako wartości całkowite jak i z-score pozostawały mniejsze w grupie urodzonych z ekstremalnie małą masą urodzeniową w porównaniu z grupą kontrolną. Przyrosty wszystkich parametrów antropometrycznych w grupie badanej w okresie między 7 a 11 rokiem życia były porównywalne do przyrostów osiąganych w grupie kontrolnej.

Parametry przemiany lipidowej u dzieci urodzonych ze skrajnie małą masą ciała nie wykazywały w naszych badaniach istotnych różnic w porównaniu ze stwierdzanymi u dzieci urodzonych o czasie z prawidłową masą ciała.

**Wnioski:**

Zarówno w siódmym jak i jedenastym roku życia dzieci urodzone z ekstremalnie małą masą urodzeniową osiągnęły mniejsze parametry wzrostowe w porównaniu z rówieśnikami urodzonymi o czasie z prawidłową masą ciała. Tempo ich wzrastania było harmonijne. Dzieci te mają również mniejszy obwód głowy. W naszych badaniach nie stwierdzono statystycznie istotnych różnic w wybranych wskaźnikach przemiany lipidowej. Konieczne są dalsze pogłębione badania rozwoju psychosomatycznego jak i metabolizmu tej grupy dzieci w celu zorganizowania najwłaściwszej wielodyscyplinarnej holistycznej opieki medycznej.

## Introduction

In recent years the survival rate of high-risk infants, especially those born prematurely with ELBW (extremely low birth weight) has increased due to extremely great progress in technological and medical sciences [1, 2, 3] but their mortality is still very high. In comparison to full-term infants, newborns with ELBW need much more multidisciplinary medical care [4, 5].

## Aim of the study

To continue the prospective follow-up cohort study on the somatic development and lipid metabolic parameters of 11-year-old children born with extremely low birth weight and to compare them with the results obtained in the study of 7-year-old children from the same group.

## Material and methods

**Fig. 1:**
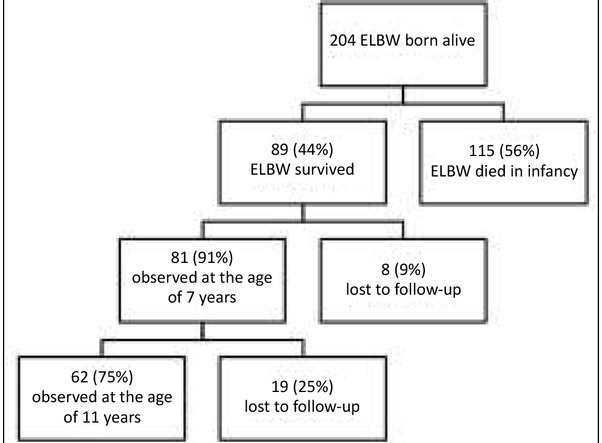
Ryc. 1. Schemat badania. Flowchart of the study.

The characteristics of 204 children included in our long-term study are shown in Figure 1. They were born with ELBW (≤1000 g) in the Malopolska voivodeship between September 2002 and August 2004. The patients were assessed twice: first between September 2009 and December 2010 at the age of 7 years (13), and the second time between December 2013 and March 2015 at the age of 11 years (fig. 1). The control group consisted of 36 children born at term that were chosen randomly from the general population and matched with regard to age and gender, and also assessed longitudinally at 7 and 11 years old. The characteristics of the subjects are listed in table I.

Anthropometric examination included the following parameters: weight (g), length/height (cm), head circumference (cm), arm circumference (cm), waist circumference (cm), and skinfold thickness at the triceps (mm); all expressed as absolute values and z-scores. We calculated the body mass index (BMI) and determined blood lipid levels for each of the study participants.

Our patients underwent all the physical measurements barefoot while wearing underclothes. Weight was taken on a medical scale to the nearest 100g. Height was measured with either a fixed stadiometer with the head set in a Frankfurt plane, or a standard medical tape to determine crown-heel length, in non-walking children to the nearest millimeter. BMI was expressed as weight in kilograms per height in meters squared. All the circumference measurements were taken by a standard medical tape to the nearest millimeter. The occipitofrontal circumference was measured over the most prominent part of the occiput and above the supraorbital ridges, the waist circumference was set at the level of the umbilicus, the arm circumference was specified at the mid-point between the olecranon process and the acromion. The skinfold thickness was evaluated with a calibrated skinfold caliper over the triceps at the midpoint between the tip of the shoulder and the tip of the elbow.

Laboratory evaluation: After the physical examination, a venous blood sample (3 ml) was taken for the assessment of serum total cholesterol, high density lipoprotein (HDL), low density lipoprotein (LDL) and triglyceride (TG) level. Total cholesterol, HDL, LDL and TG were measured by a colorimetric method, using the VITROS Integrated System (Ortho Clinical Diagnostics, New Jersey, USA). Age-adjusted normal reference ranges were used to assess the accurate level of the lipid profile. Normal values [mmol/l] were as follows: total cholesterol 2.95-5.57, HDL 0.69-1.81, LDL 1.51-3.34 TG 0.57-2.36 for girls, and total cholesterol 2.82-5.27, HDL 0.65-1.81, LDL 1.27-3.35 TG 0.51-2.12 for boys.

Statistical analysis was conducted using JMP 9.0.0, 2010 SAS Institute Inc. The detected differences were considered to be statistically significant in case of p<0.05. Demographic and clinical data comparisons between the study and control groups were performed using Student’s t-test, Mann-Whitney U-test, chi-square test. The comparisons between the measurements acquired at 7 and at 11 years were performed using the paired t-test.

The protocol of the study was approved by the Jagiellonian University Medical College Ethics Committee. The parents of all the participants provided written informed consents.

## Results

A total of 89 children born with ELBW were enrolled in the study, 81 were evaluated in their 7^th^ year of life, and 62 children − in their 11^th^ year of life (Fig. 1). The control group consisted of 36 children born at term that were chosen randomly from the general population and matched with regard to age and gender, and also assessed longitudinally. Characteristics of the subjects are listed in Table I.

**Table I:** Baseline characteristics of the study and control population (mean values and standard deviation). Tabela I. Wyjściowa charakterystyka grupy badanej i grupy kontrolnej (dane wyrażone jako średnia i odchylenie standardowe).

	Study group *Grupa badana*	Control group *Grupa kontrolna*	p-value *Wartość p*
Gestational age (wk.) *Wiek* *płodowy* *(tyg.)*	27.3 (2.3)	39.8 (1.4)	<0.0001* (1)
Birth weight (g) *Urodzeniowa masa ciała (g)*	843.4(132.4)	3589.4(538.8)	<0.0001* (1)
Gender(boys/girls) *Płeć (męska/żeńska* )	21/41	19/17	n.s.
Cesarean section *Poród drogą cięcia cesarskiego*	43	5	<0.0001* (2)
Infant Small for Gestational Age *Noworodek za mały do wieku płodowego*	19	2	0.0045* (2)
Newborn – from 1^st^ pregnancy *Dziecko z* *ciąży* *pierwszej*	23	23	0,048* (2)
Age at 1^st^ assessment (year) *Wiek w czasie 1-szego badania (lat)*	6.61 (0.36)	6.98 (0.83)	0.03* (1)
Age at 2^nd^ assessment (year) *Wiek w czasie 2-giego badania (lat)*	11.06 (0.38)	10.62 (0.82)	0.0004* (1)

*p-value for: t-Student test (1), Chi-square test (2),

At 7 years, children (13) who were born as ELBW preemies were shorter (113.75 cm (-0.72) vs.124.52 cm(0.53)), lighter (19.47 kg (-1.12) vs. 25.23kg (0.39)), had smaller head circumference (49.81 cm (-2.19) vs. 52.5cm (-0.377)), reduced waist circumference (50.14 cm (-0.83) vs. 55.45 cm (0.34)), reduced middle upper arm circumference, MUAC (17.51 cm, v. 19.29 cm), reduced skinfold thickness (0.76 cm (-0.817) vs. 0.92 cm (-0.19)) and smaller BMI (14.5 (-0.99) vs. 16.16 (0.12)) expressed both as absolute values and z-score values compared to the control group (tab. II).

**Table II:** Analysis of the anthropometric parameters and their comparison between the study group and the control group at 7 years old (mean values and standard deviation). Tabela II. Analiza parametrów antropometrycznych ocenianych w wieku 7 lat w grupie badanej i kontrolnej (dane wyrażone jako średnia i odchylenie standardowe).

	Study group *Grupa badana*	Control group *Grupa kontrolna*	p-value *Wartość p*
Height (cm) *Wysokość (cm)*	113.749 (15.66)	124.52 (7.39)	<0.0001* (1)
Height z-score *Wysokość (z-score)*	-0.722 (1.215)	0.532 (0.934)	<0.0001* (1)
Weight (kg) *Masa ciała (kg)*	19.47 (3.85)	25.23 (5.35)	<0.0001* (1)
Weight z-score *Masa ciała* *(z-score)*	-1.123 (1.38)	0.39 (1. 186)	<0.0001* (1)
Head circumference (cm) *Obwód głowy (cm)*	49.81 (1.72)	52.5 (1.33)	<0.0001* (1)
Head circumference z-score *Obwód* *głowy z-score*	-2.19 (1.33)	-0.377 (0.912)	<0.0001* (1)
MUAC (cm)	17.51 (1.97)	19.29 (2.675)	0.0009* (1)
Skinfold thickness at triceps (cm) *Grubość fałdu nad mięsniem trójgłowym*	0.76 (0.29)	0.92 (0.3)	0.0144* (1)
Skinfold thickness at triceps z-score *Grubość fałdu nad mięsniem trójgłowym z-score*	-0.817 (0.98)	-0.191 (1.08)	0.0065* (1)
Waist circumference (cm) *Obwód* *pasa (cm)*	50.14 (4.75)	55.45 (6.56)	0.0002* (1)
Waist circumference z-score *Obwód pasa z-score*	-0.836 (1.314)	0.34 (1.38)	0.0006* (1)
BMI (kg/m^2^)	14.5 (1.98)	16.16 (2.34)	0.0007* (1)
BMI z-score	-0.999 (1.465)	0.12 (1.334)	0.0002* (1)

*p-values for Student’s t-test (1); MUAC − mid-upper arm circumference/*obwód ramieni*a, *BMI – body mass index/*wskaźnik masy ciała B*MI

At 11 years old, the ELBW children were still smaller than their born-at-term peers as demonstrated by the anthropometric parameters such as height (141.7 cm (-0.368) vs. 146.26 cm (0.65)), weight (33.88 kg (-0.59) vs. 40.45 kg (0.66)), head circumference (51.37 cm (-2.05) vs. 54.02 cm (-0.33)), waist circumference (61.7 cm (0.26) vs. 67.84 cm (1.06), MUAC (20.95 cm, vs. 22.85 cm), skinfold thickness (1.17 cm (-0.25) vs. 1.68 cm (0.78)) and BMI (16.74 (-0.62) vs. 18.72 (0.36)) expressed both as absolute values and z-score values (tabl. III).

**Table III:** Analysis of anthropometric parameters and their comparison between the study group and control group at 11 years old. (mean values and standard deviation). Tabela III. Analiza parametrów antropometrycznych ocenianych w wieku 11 lat w grupie badanej i kontrolnej (dane wyrażone jako średnia i odchylenie standardowe).

	Study group *Grupa badana*	Control group *Grupa kontrolna*	p-value *Wartość p*
Height (cm) *Wysokość (cm)*	141.7 (7.83)	146.26 (8.81)	0.0118* (1)
Height z-score *Wysokość (z-score)*	-0.368 (1.135)	0.65 (1.081)	<0.0001* (1)
Weight (kg) *Masa ciała (kg)*	33.88 (8.315)	40.45 (9.96)	0.0013* (1)
Weight z-score *Masa ciała (z-score)*	-0.592 (1.4)	0.6634 (1.2)	<0.0001* (1)
Head circumference (cm) *Obwód* *głowy (cm)*	51.37 (2.24)	54.02 (1.42)	<0.0001* (1)
Head circumference z-score *Obwód* *głowy z-score*	-2.05 (1.63)	-0.33 (0.86)	<0.0001* (1)
MUAC (cm)	20.95 (2.96)	22.85 (3.66)	0.0097* (1)
Skinfold thickness at triceps (cm) *Grubość fałdu nad mięśniem trójgłowym*	1.172 (0.52)	1.683 (0.7)	0.0003* (1)
Skinfold thickness at triceps z-score *Grubość fałdu nad mięśniem trójgłowym z-score*	-0.253 (1.15)	0.786 (1.123)	<0.0001* (1)
Waist circumference (cm) *Obwód pasa (cm)*	61.7 (8.95)	67.,84 (11.14)	0.0063* (1)
Waist circumference z-score *Obwód pasa z-score*	0.264 (1.5)	1.064 (1.41)	0.0126* (1)
BMI (kg/m^2^)	16.74 (3.26)	18.72 (3.4)	0.0058* (1)
BMI z-score	-0.628 (1.6)	0.364 (1.37)	0.0023* (1)

*p-values for Student’s t-test (1); MUAC − mid-upper arm circumference/*obwód ram*ienia *BMI – body mass index/*wskaźnik masy ciała BMI*

The anthropometric parameters analyzed in the study and control groups increased at comparable rates along their estimated channels of the growth chart, with exception of height, which was significantly faster among the ELBW children (tab. IV).

**Table IV:** Comparison of changes in values of anthropometric parameters taken at first at 7, and then at 11 years old in the study and control groups (mean values and standard deviation). Tabela IV. Porównanie zmian parametrów antropometrycznych w okresie między 7 a 11 rokiem życia w grupie badanej i kontrolnej (dane wyrażone jako średnia i odchylenie standardowe).

	Difference between 1^st^ and 2^nd^ examination - study group *Różnica pomiaru pomiędzy pierwszym a drugim badaniem - grupa badana*	Difference between 1^st^ and 2^nd^ examination - control group *Różnica pomiaru pomiędzy pierwszym a drugim badaniem - grupa kontrolna*	p-value Wartość p
Height (cm) *Wysokość (cm)*	27.95 (15.35)	21.74 (3.52)	0.0026 *(1)
Height z-score *Wysokość (z-score)*	0.35 (0.68)	0.12 (0.33)	0.0265*(1)
Weight (kg) *Masa ciała (kg)*	14.2 (6.26)	15.2 (5.74)	0.4195 (1)
Weight z-score *Masa ciała (z-score)*	0.5 (1.02)	0.24 (0.58)	0.1118(1)
Head circumference (cm) *Obwód* *głowy (cm)*	1.54 (1.38)	1.51 (0.71)	0.9 (1)
Head circumference z-score *Obwód głowy z-score*	0.117 (1.09)	0.158 (0.44)	0.7934 (1)
MUAC (cm)	3.39 (2.16)	3.56 (2.73)	0.7466 (1)
Skinfold thickness at triceps (cm) *Grubość fałdu nad mięśniem trójgłowym*	0.41 (0.05)	0.77 (0.57)	0.0017 *(1)
Skinfold thickness at triceps z-score *Grubość fałdu nad mięśniemtrójgłowym z-score*	0.56 (0.97)	1.01 (1.03)	0.0393* (1)
Waist circumference (cm) *Obwód* *pasa (cm)*	10.23 (5.84)	12.39 (7.35)	0.1748 (1)
Waist circumference z-score *Obwód* *pasa z-score*	1.03 (1.13)	0.91 (0.165)	0.6421 (1)
BMI (kg/m^2^)	2.14 (2.19)	2.64 (1.71)	0.215 (1)
BMI z-score	0.34 (1.07)	0.42 (0.77)	0.71 (1)

*p-values for Student’s t-test (1); MUAC − mid-upper arm circumference/*obwód ramienia*, *BMI – body mass index/*wskaźnik masy ciała BMI*

We noted that ELBW male subjects were significantly smaller, as shown in all the recorded physical growth parameters, compared to their matched controls at 11 years, while ELBW females demonstrated physical growth (such as height, weight, MUAC, waist circumference and BMI) comparable to their controls at 11 years (data not shown). There was no statistically significant difference with regard to lipid profile between the two investigated groups (tab. V).

**Table V:** Analysis of changes in lipid profile in period of time between 7 and 11 years old. Tabela V. Analiza zmian profilu lipidowego w okresie między 7 a 11 rokiem życia w grupie badanej i kontrolnej.

	Difference between 1^st^ and 2^nd^ examination - study group *Różnica pomiaru pomiędzy pierwszym a drugim badaniem - grupa badana*	Difference between 1^st^ and 2^nd^ examination - control group *Różnica pomiaru pomiędzy pierwszym a drugim badaniem - grupa kontrolna*	p-value
LDL (mmol/l)	-0.25	-0.24	0.9140 (1)
HDL (mmol/l)	0.016	0.08	0.5500 (1)
TG (mmol/l)	-0.004	0.16	0.3000 (1)
Total Cholesterol (mmol/l)	-0.4	-0.13	0.0530 (1)

p-value for Student’s t-test (1). LDL − low density lipoproteins, HDL − high density lipoproteins, TG − triglycerides. LDL – lipoproteiny niskiej gęstości (low density lipoproteins), HDL – lipoproteiny wysokiej gęstości (high density lipoproteins), TG – trójglicerydy (triglycerides).

## Discussion

We present the results of a longitudinal prospective cohort study that explores potential correlations between birth weight, postnatal weight gain and anthropometric variables of very preterm-born children once they reach the age of 11 years. The study showed that ELBW preterm children demonstrated much poorer growth than full-term children at 11 years. The stature of our controls very closely matched that of the normal population, unlike the ELBW children whose mean height was significantly lower. The mean deficit in stature in the ELBW group was around 4.6 cm, and it was comparable to the height difference found by Peralta- Carcelen and colleagues in their study of 14-year-old VLBW (very low birth weight) children [8]. Saigal et al. [9] showed that children born ELBW were at an increased risk for short stature in adolescence. Not only were the preterm children shorter and lighter, but they also had relatively smaller head circumferences. We presented similar results, our ELBW children’s head circumferences were significantly smaller (-2 SD) compared to the reference values. We hypothesized that those children might eventually develop lower IQ, since there were reports of a correlation between head circumference and IQ (10). We used Z-scores in our study to monitor changes during the development of our ELBW children. We believe that the results reported in such a way added more strength to our longitudinal study, since according to Shann, the use of Z-scores comparable across ages provided a more sensitive assessment of deviations of growth than the use of percentiles [11].

We examined some indices of lipid metabolism since preterm infants might have an increased risk of adverse metabolic outcomes in later life. We also checked the metabolic status of our subjects by analysis of their fat mass and lipid profile. We measured their mid-upper arm circumference, skinfold thickness and waist circumference, although we did not assess the amount of fat mass directly using the DXA method, which is known to be a gold standard. Anthropometric parameters were significantly lower in comparison to the control group both at 7 and 11 years of age. Our results were similar to the findings of Gianni et al. [12], who examined children with birth weight <1800 g and gestational age <34 weeks at the age of 4.8-6.6 years using dual energy x-ray absorptiometry (DXA).They found a significantly lower amount of adipose tissue in the group of preterm infants in comparison with the full-term infants’ group. Moreover, they noted aberrant fat distribution with reduced subcutaneous and increased intra-abdominal amount of adipose tissue. However, we observed a “catch-up” period in our ELBW children during the time-span of our study. When we analyzed the z-score values, their skinfold thickness and waist circumference were both at -0.8SD at 7 years, and increased to -0.2 and +0.2 at 11 years, respectively. It was in agreement with the work by Hack M. et al., who carried out a prospective cohort study of VLBW infants.They noted that the catch-up growth in weight, height, and BMI occurred between 8 and 20 years among VLBW females, in contrast to the VLBW males who remained significantly smaller than their controls at 20 years old [6, 7]. In another study, Peralta-Carcelen et al. compared the growth parameters between a group of almost 15-year-old ELBW adolescents and their sex and age matched controls born at normal birth weight (NBW). On average, ELBW adolescents were shorter and lighter than their normal-birth-weight NBW counterparts. Just as in all the other studies, the ELBW adolescents had lower mean z-scores for height, weight, and head circumference than NBW adolescents. However, in agreement with our findings, their body composition was similar between groups [8].

We believe that our study further emphasizes the need for a careful follow-up through the adolescent years of ELBW children. Further research is needed to fully understand long-term metabolic and psychosomatic developmental implications with a holistic [14] approach to the therapy.

## Conclusions

Preterm extremely low birth weight (<1000 g) children are at an increased risk of growth failure. Once they reach teenage years, they are shorter and lighter than their age- and sex-matched peers born-at-term. They also have smaller heads. The indices of lipid metabolism we selected did not show statistically evident differences between groups. There is a need for longitudinal studies to further monitor the development of children born with extremely low birth weight in order to organize proper interdisciplinary medical care with a holistic approach to the therapy [14].
